# CustusX: an open-source research platform for image-guided therapy

**DOI:** 10.1007/s11548-015-1292-0

**Published:** 2015-09-26

**Authors:** Christian Askeland, Ole Vegard Solberg, Janne Beate Lervik Bakeng, Ingerid Reinertsen, Geir Arne Tangen, Erlend Fagertun Hofstad, Daniel Høyer Iversen, Cecilie Våpenstad, Tormod Selbekk, Thomas Langø, Toril A. Nagelhus Hernes, Håkon Olav Leira, Geirmund Unsgård, Frank Lindseth

**Affiliations:** Department of Medical Technology, SINTEF Technology and Society, Trondheim, Norway; Norwegian University of Science and Technology (NTNU), Trondheim, Norway; Norwegian National Advisory Unit on Ultrasound and Image-Guided Therapy, St. Olavs Hospital - Trondheim University Hospital, Trondheim, Norway

**Keywords:** Image-guided therapy, Intraoperative ultrasound, Open source, Platform, Computer-assisted interventions

## Abstract

**Purpose:**

CustusX is an image-guided therapy (IGT) research platform dedicated to intraoperative navigation and ultrasound imaging. In this paper, we present CustusX as a robust, accurate, and extensible platform with full access to data and algorithms and show examples of application in technological and clinical IGT research.

**Methods:**

CustusX has been developed continuously for more than 15 years based on requirements from clinical and technological researchers within the framework of a well-defined software quality process. The platform was designed as a layered architecture with plugins based on the CTK/OSGi framework, a superbuild that manages dependencies and features supporting the IGT workflow. We describe the use of the system in several different clinical settings and characterize major aspects of the system such as accuracy, frame rate, and latency.

**Results:**

The validation experiments show a navigation system accuracy of $<$1.1 mm, a frame rate of 20 fps, and latency of 285 ms for a typical setup. The current platform is extensible, user-friendly and has a streamlined architecture and quality process. CustusX has successfully been used for IGT research in neurosurgery, laparoscopic surgery, vascular surgery, and bronchoscopy.

**Conclusions:**

CustusX is now a mature research platform for intraoperative navigation and ultrasound imaging and is ready for use by the IGT research community. CustusX is open-source and freely available at http://www.custusx.org.

## Introduction

In image-guided therapy (IGT), the surgeon uses medical images spatially registered to the patient together with computer tracked instruments to plan and guide the surgical procedure. The basic principles of image guidance have been widely adopted and are commercialized through a number of platforms such as Medtronic’s StealthStation, Brainlab’s Curve system, and Sonowand Invite for neurosurgery, Covidien’s superDimension for interventional lung procedures, Karl Storz’ NBU system for ear–nose–throat procedures, Siemens’s Orbic system for orthopedic, trauma, and spine procedures, and others. These systems are in general closed systems, and their image data, position/tracking data, algorithms, and visualization methods are not accessible for independent research groups. In order to perform research, development, and validation in the field of IGT, systems with open access to the system’s data and methods are crucial. Several open access research systems have been made available to the medical and research communities over the last ten years. 3D Slicer [1] was initiated in 1998 and was rearchitected and released in 2007. 3D Slicer supports multimodal images and is interfaced to multiple widely used toolkits in the field of medical imaging and navigation. It also has a plugin system for anyone who wants to add new features to the system. With the addition of IGTLink and PLUS [2] (public software library for ultrasound imaging research), 3D Slicer can also provide integrated navigation and ultrasound imaging. Another platform is MITK [3] (Medical Imaging Interaction Toolkit) with IGT modules and plugins. Yet another system is IBIS [4], a closed-source research system with integrated ultrasound imaging dedicated to neurosurgery.

CustusX, which we present in this paper, is an open-source research platform with focus on intraoperative navigation and ultrasound (US) imaging. CustusX was originally initiated in 1998 by the Norwegian National Advisory Unit for Ultrasound and Image-Guided Therapy,^1^ Trondheim, Norway, and has been continuously developed, tested, and adapted for more than 15 years, and completely rearchitected in 2007. CustusX has been developed in close collaboration between software developers, technical and medical researchers and has been used in a number of scientific studies covering a wide range of clinical applications. The system has recently been released^2^ as an open-source software to the research community as part of the ongoing open science effort in the scientific and software communities [5]. The chosen license is BSD, meaning that the software can by used by everyone for anything, including commercial use. CustusX is also the basis for further development of the IGT part of the national (Norwegian) research platform NorMIT.^3^ In the following chapter, CustusX is presented in terms of overall structure, software architecture, and key concepts of workflow. The software quality process is described in “Software quality process” section. “Navigation system accuracy and system response” section describes a series of accuracy and responsiveness experiments characterizing the main components of the system, and finally a series of clinical applications are presented in “Clinical applications” section. A discussion of the main strengths and limitations of the system and directions of future development concludes the paper.

## System description

The users of CustusX are typically a mix of clinical and technological researchers. Based on this, we can formulate a set of requirements which in turn determine the architecture and features.

### Requirements

As an IGT platform, CustusX should be focused toward research and clinical trials in the operating room (OR). The purpose of the system should be to aid the users during the clinical procedure, from planning through intraoperative guidance to postoperative control. Components used during therapy, such as visualization and navigation, should be made with the intention of being simple, intuitive and for the most part automated. Pre- and postoperative features may be limited, and such features can instead be covered by good interoperability with other image analysis tools. The system should be robust, i.e., be up and running during the entire clinical procedure, and it must deliver functionality within the limited time frame available in the OR.

From the developer point of view, the platform must be easy to extend while retaining a stable core. Unstable extensions must not impede the stability of the rest of the system. The platform should support specific clinical procedures and present a customized user interface when used in the OR.

### Overall structure and user interface

**Fig. 1:**
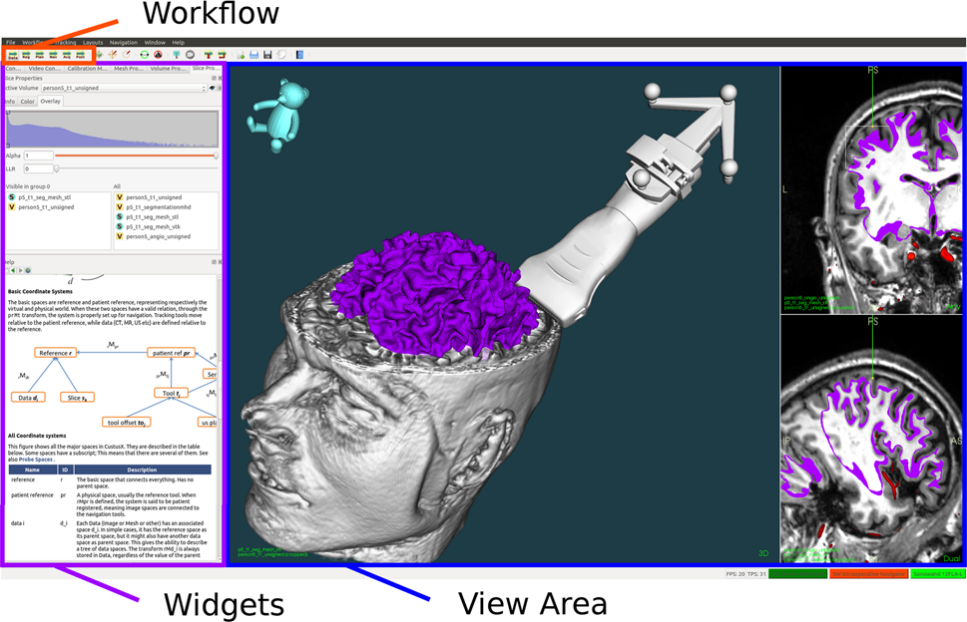
Main window. The virtual patient is shown in the central view area, using a combination of 2D and 3D views. Other user interaction components such as widgets and toolbars are also available

CustusX uses a main window user interface based on the Qt QMainWindow class, which provides menus, toolbars, configurable widgets, and a central view area, as shown in Fig. 1. During navigation, the view area is where the clinician interacts with the patient using surgical tools, pointers and US probes. The main window is highly customizable, and a specific setup can be stored for later use.

A clinical procedure (e.g., neurosurgery, endovascular interventions, laparoscopy) is modeled as a workflow, consisting of several discrete steps. Each step involves its own operations and visualization.

The user moves between these steps during the procedure, and each one has an associated custom user interface that again can be personalized by storing the main window setup. As the customization of the graphical user interface (GUI) can be done beforehand, this minimizes the need for setup in the OR.

### Architecture and software components

**Fig. 2:**
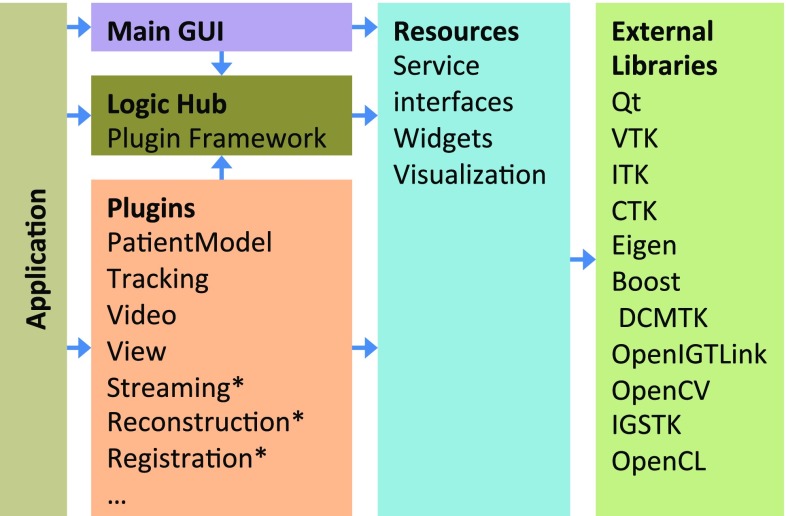
CustusX architecture. *Arrows* show the dependency direction. The application initialize the plugins and the user interface, with the Logic Hub as a plugin repository. Plugins contribute to functionality and extensibility in a modular way. The plugins marked with an *asterisk* have multiple instances. Resources and components from external libraries are used as building blocks throughout the system

CustusX uses a layered architecture, as shown in Fig. 2. The external libraries contribute to much of the functionality, with CustusX being an integrating platform. The resource layer provides what cannot be found externally, in addition to defining service interfaces. Plugins contain the majority of the code and provide discrete chunks of features on a high level of abstraction.

#### External libraries

Qt^4^ provides a crossplatform GUI/application framework and is used throughout the application, at the same level as the C++ libraries. Qt simplifies C++ development through its signals and slots mechanism and extensive libraries. It is used in many large open-source projects and thus has a long expected lifetime. VTK^5^ and ITK^6^ are used for visualization, registration, and image processing, CTK [3] for plugin and DICOM support, Eigen^7^ for basic math, while DCMTK,^8^ OpenCV [6], OpenIGTLink [7], IGSTK [8], PLUS, and others are used for specialized operations.

#### Superbuild

To maintain an application with dependencies to other large libraries is non-trivial. Operating systems and libraries evolve at different speeds and are often incompatible. The solution for this problem is to create a superbuild that downloads specific versions of each library and builds them for the application in question.

The CustusX superbuild is implemented using a Python layer on top of CMake. It downloads, configures, and builds CustusX and the libraries it depends on, and then tests and deploys resulting artifacts on all major desktop platforms. The Python layer gives added flexibility but can be unfamiliar. Another solution used by several other projects (e.g., 3D Slicer, MITK, CTK) is to base the superbuild on the CMake ExternalProject module and use only CMake.

#### CTK Plugin Framework

CustusX uses the OSGi-based [9] CTK Plugin Framework [3] as the basis for most of the architecture after having converted from a homegrown plugin system in 2014. This framework introduces two central concepts: services and plugins (OSGi uses the name bundle instead of plugin). Services are implementations of abstract interfaces available from the central plugin framework hub. Service consumers are unaware of the service providers. Services may also appear and disappear at any time. Plugins are physical components (shared libraries) that can be loaded and unloaded at runtime. Each plugin provides one or more service implementations, while they also can act as service consumers.

#### Services

An IGT platform requires some core services in order to operate. These are declared as interfaces in the resource layer, thus making them available to everyone. They are implemented in plugins that are always included with the system, and we assume there is one instance of each:

- *Patient model* A model describing the virtual patient.
- *Tracking* Connection to tracking systems.
- *Video* Access to video sources and US scanners.
- *View* Visualization and navigation.
- *Acquisition* Record real-time data.
- *Registration* Framework for registration methods.
- *Reconstruction* Framework for US reconstruction methods.

Services can also be used as extension points, i.e., adding more instances of the same service adds more functionality to the system. The following extension points are available:

- *GUI extender* Provides a collection of widgets and toolbars that can be added to, for example, the main window. This is the most generic extension point and can be used for anything that can be accessed from a GUI.
- *Registration method* Defines a registration method that is made available through the registration service.
- *US Reconstruction method* Defines a US reconstruction method that is made available through the US reconstruction service.
- *Streamer* Supports a video source or a US scanner, used by the video service.
- *Filter* Defines an algorithm that can be applied to volume or geometric data.
- *Tracking system* Supports a specific tracking system.

#### Extensibility

The plugin framework is an excellent way to create a modular system. It is also useful for developers who want to extend the system with their own features. By creating a plugin that implements some of the extension point services, the feature is automatically added to the build system.

Plugins can be used to incorporate code that is proprietary for some reason and thus cannot be released as open source. The proprietary plugins can be contained in separate repositories and added to CustusX using a superbuild script.

The main window is the standard front end for CustusX. However, it is possible to create or use another front end and rather use CustusX as a toolkit for creating custom applications.

### Workflow

**Fig. 3:**
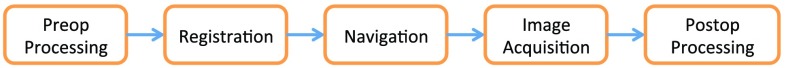
A normalized clinical workflow as used by CustusX. The steps model a procedure from data preparation and registration through intraoperative navigation and image acquisition, ending in postoperative analysis

As mentioned earlier, in CustusX the clinical procedure is modeled as a workflow dividable into a sequence of steps, as shown in Fig. 3. The following sections describe features that are related to each of the workflow steps. As CustusX is dedicated to IGT, this is also the most important features of the system.

#### Preoperative processing

Preoperative processing is the preparation of images through image processing and the generation of the *patient model*, which is a virtual model of the patient containing volume data, geometric models, and spatial and temporal relations between these. DICOM, MetaImage,^9^ vtk, and stl file formats can be imported directly. Image preprocessing (e.g., segmentation and surface generation) can be done using a variety of tools, e.g., OsiriX [10], ITK-SNAP [11], or 3D Slicer [1], prior to import into CustusX.

Furthermore, CustusX contains some image processing methods that also have been found useful intraoperatively. One such example is the extraction of tubular structures, which is used for both bronchiae and vascular structures. Two methods for this are currently included: A binary thinning method [12] and a segmentation-based method [13].

#### Registration

Registration is the process of determining the spatial relation between two coordinate systems. The standard relation is a rigid transformation, although nonlinear relations also exist. There are two general forms:

*Image to image* (I2I) registers one (moving) set of images to another (fixed), effectively moving one set into the coordinate space of the fixed. The ITK Software Guide [14] describes I2I registration in more detail.

*Image to patient* (I2P) registers the virtual patient to the physical, thus enabling image navigation using physical tools. I2P can be viewed as a variant of I2I, with the fixed image replaced with the physical space.

Validation is done like this: (a) visually by undoing/redoing the registration and inspecting the difference, (b) quantitatively by defining landmarks in the moving space and measuring the movement imposed by the registration.

Specific registration methods are implemented as services:

*Landmark registration method* works by defining the same point set in the two spaces to be registered and then finding the relation between them using a least squares fit as implemented by VTK. It can be used for both I2I and I2P registration.

*Fast registration method* is a variant of the I2P landmark registration. The orientation of the tool in addition to defining at least one landmark is used to find a rough registration. This is not an accurate method, but is fast and can be used as an initial estimation for more refined methods.

*Centerline registration method* performs I2I registration by minimizing the distance between two curved paths using an iterative closest point (ICP) algorithm [15].

*Command-line interface* can be used to access any I2I registration process in external tools. ElastiX [16] and ImFusion [17] are examples of such tools.

#### Navigation

Navigation is the main focus in an IGT system, where the purpose is to use information from different imaging modalities as an accurate guidance map, visualized to the clinical operators during procedures. Position tracking technology is used to dynamically visualize instruments and tools at the correct location in the 3D guidance map.

*Tracking* Tools are physical devices that are used by the operator to interact with the patient. Examples of tools are any surgical instruments, pointers, US probes, and the computer mouse. The IGSTK library is used to connect to the tracking systems, providing support for both optical—(Polaris, NDI, Canada) and electromagnetic (Aurora, NDI, Canada) tracking. Sensors are placed on the instruments, and the tracking system provides position and orientation data for the sensors in real time. The matrix describing the tool tip position relative to the sensors is then obtained from the device supplier or found using a calibration procedure [18]. Tool configurations can be setup from the GUI and are stored in xml files. Once set up, tracking of the configured tools automatically starts at system startup.

*Views* The patient model and the tools are visualized in a collection of 2D and 3D Views. Standard functionality from VTK is used to provide rendering of multiple volumes in 3D, using preset or user-defined transfer functions. A fast custom image fusion algorithm is used to provide overlayed 2D images. The views can be displayed in various configurations on multiple screens, giving, e.g., the clinician a dedicated screen containing only visualizations, while the researcher can use a more technical screen.

*Navigation* The clinician navigates through the patient anatomy using tools, and the views show the position inside the virtual patient. The default method is to visualize a fixed patient with a moving tool, using the axial, coronal, and sagittal planes in the 2D views. An alternative is the tool view, which uses a fixed camera and a moving patient and can be more intuitive during, e.g., an endoscopic procedure. This is available in 3D, where the camera is placed on the tool tip, and in 2D, where the slice planes are attached to the tool tip.

#### Image acquisition

During the procedure, there might be a need to acquire updated image data, because of patient movement, anatomical changes, or lack of preoperative data. CustusX can use image modalities such as ultrasound, endoscopic video, or cone beam CT to provide this updated information. With a properly calibrated and tracked US probe, new 3D volumes can be created.

*US scanner connection* Several options for connecting an US scanner and receiving US scanner video signals are available. The basic solution that works for all scanners is a video grabber (DVI2USB 3.0, Ephiphan, USA) via OpenCV. Digital video streaming is a possibility with US scanners providing an application programming interface (API). Currently, CustusX connects to the API of Ultrasonix MDP (Analogic Ultrasound, Canada) and Vivid E9 (GE Healthcare, USA), the Vivid E9 connection being a proprietary plugin. While the video grabber only supports one 2D stream, the API connections allow for multiple streams (such as tissue and flow images), 3D streams, and probe configuration information.

**Fig. 4:**
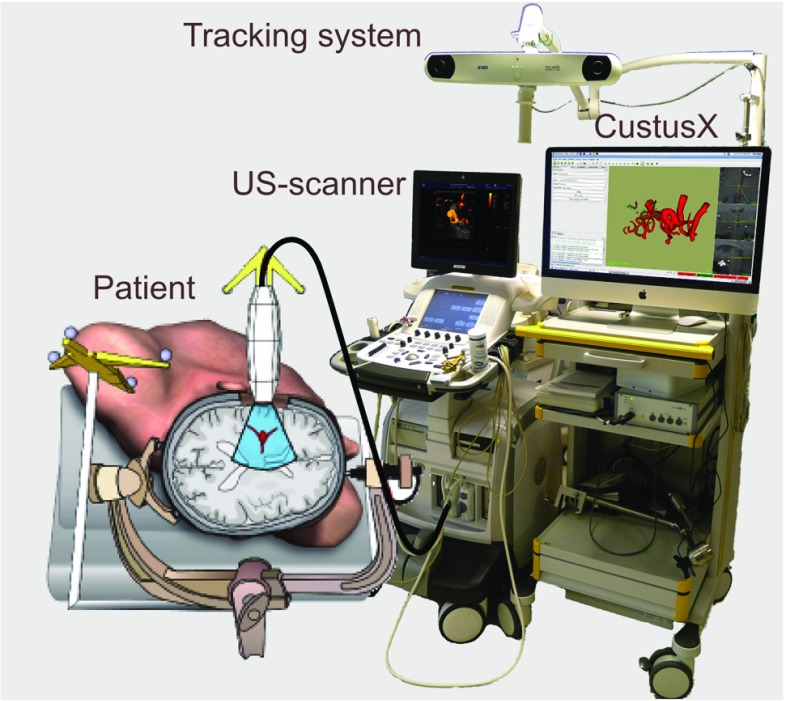
An example setup of CustusX for use in neurosurgery, with a tracked US probe imaging the patient

*US probes* US video streams are calibrated to the tracking system, both with spatial and temporal calibration [18]. This allows CustusX to position and visualize the video stream at the correct position and orientation in the patient model. The US probe configuration, such as width and depth, is also needed so that invalid image parts can be clipped away. The configuration can be obtained automatically from scanners with an API connection, but must be supplied manually when using video grabbers. Figure 4 shows an example setup.

**Fig. 5:**
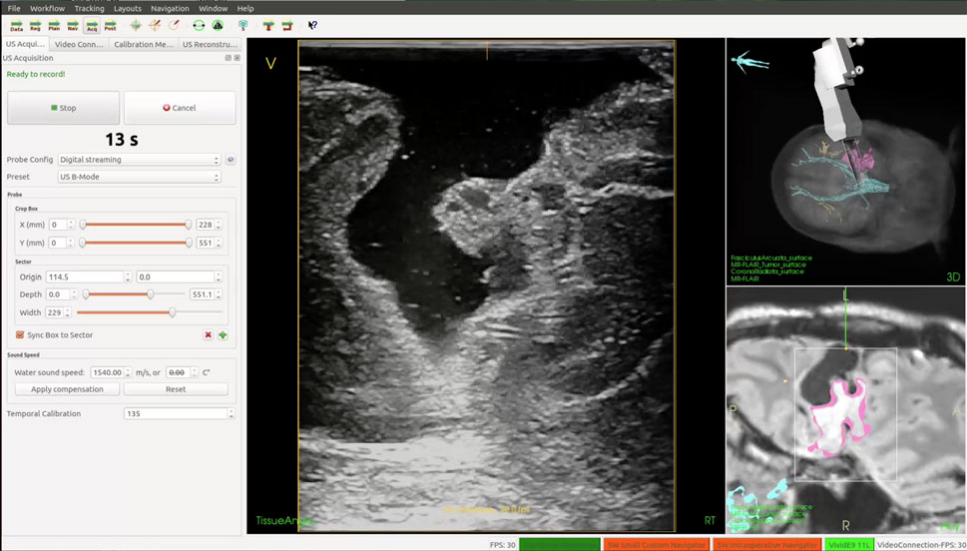
Ultrasound acquisition during brain tumor surgery

*3D US reconstruction* A 3D US volume can be reconstructed from a freehand recording of a 2D US image stream from a properly configured US probe, by mapping all the stream data into a single volume. These reconstructions are done either on the CPU [19], or on the GPU [20]. Figure 5 shows CustusX during a freehand recording. The resulting volume can be visualized to give updated 3D information to the clinician.

#### Postoperative processing

The data generated during a procedure can be analyzed through postoperative processing and stored for later use. CustusX provides a few tools for this:

*Measurement* Points, planes, distances, and angles can be defined and shown in the 3D scene. This can be used for measurement, planning, or annotation.

*Recording and playback* The entire session history can be stored for later use. This includes tool movement, registration events, and video recordings. A special playback mode can be used to inspect the stored session at any point in time, but with the option to change the camera angle, zoom, and views. Alternatively, the entire session can be recorded as a movie using an external application.^10^

## Software quality process

The CustusX IGT platform is designed to be used in research involving patients. This requires that the quality and stability of the software are high, even if its use is only within the context of research and the clinicians never depend on the platform to make decisions. All use on patients requires approval by an ethics board or similar for each specific study. The standards for medical software such as IEC 62304 are not supported. Quality also is important from a software engineering point of view: Feature production should be maximized as opposed to maintenance and bug fixing, and it is equally important that the right features are implemented. The CustusX quality process addresses these issues.

### Development process

The planning of CustusX development is done in cooperation between *clinical researchers*, *technological researchers*, and *developers*, who cooperate closely, both by discussions, demonstrations, and by developers attending clinical procedures. Biannual planning sessions attended by all three parties determine the major features to implement. The work is then done in iterations allowing for corrections along the way, thus following an agile process [21]. The developers use techniques such as pair programming, daily standup, unit testing, and continuous refactoring to increase quality. Some of these techniques are only available because the core team is situated in the same location. With the recent conversion to open source, this will be augmented with a distributed process using github^11^ as a basis.

### Continuous integration

**Fig. 6:**
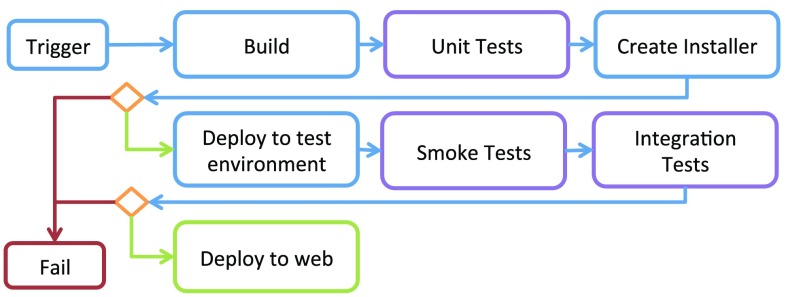
Continuous integration flow. The flow is triggered by either a commit, a nightly timer, or a manual request. The system is then built and tested in both the build folder and after deployment. Failure at any stage aborts the flow. If all steps succeed, the binaries are published on a Web server

In order to get feedback from the tests as quickly as possible, a continuous integration methodology is used [22]. This requires that the build system is fully automated and that a suite of tests are in place. Every commit made to the main git branch is picked up by a Jenkins server [23], then built and tested on all target platforms (currently Windows 8, Ubuntu 14.04, and MacOSX 10.8). Because of the sensitivity with regard to choice of hardware such as GPU, the tests are performed on physical machines similar to those used in the OR. Figure 6 shows the test flow.

The Python superbuild framework is used to perform these operations. Catch^12^ is used to run unit tests, all of which are run as a single process in order to maximize speed. CTest is used to run integration tests, as they usually require each test to be run as a stand-alone process. Smoke tests involve starting all executables to validate the deployment.

A dashboard gives feedback on which tests that failed or passed. In addition, we have created extreme feedback devices [23] in the shape of red or green gummy bear lamps reporting the status to the developers in a very visual way.

### Release cycle

The CustusX project operates with three types of releases: alpha, beta, and full. The difference is mainly the level of manual testing involved. Automated tests give a basic confidence, while structured manual tests give more complete coverage on the system level.

*Alpha releases* are products of the continuous integration system. They are produced automatically, but no manual testing is involved.

*Beta releases* undergo a basic manual test, often with a focus on a small set of features that is needed at that moment.

*Full releases* have undergone a full system test and stabilization period. The system test is a written document supplementing the automated test, intended to catch errors not easily automatable. There are about 2 full releases each year.

All releases are one-click operations. The alpha releases can be retrieved from the build server, while beta and full releases are created using a Python script that attaches the correct version tag, runs the Jenkins tests, and moves the installer to a release server.

## Navigation system accuracy and system response

The delicacy, precision, and extent of the work the surgeons can perform based on the image information provided rely on their confidence in the overall clinical accuracy, robustness, and responsiveness of the system and the anatomical or pathological representation of relevant structures.

### Navigation system accuracy

The overall clinical accuracy in IGT is often referred to as the navigation system accuracy (NSA) and is defined as the difference between the location of a surgical tool relative to some anatomical structure as displayed in the image information and the true physical location relative to the same structure within the patient. This accuracy is difficult to assess in a clinical setting due to the lack of fixed and well-defined landmarks inside the patient that can be accurately reached with a pointer. Common practice is therefore to estimate the system’s overall accuracy in a controlled laboratory setting using accurately built phantoms. In order to conclude on the potential clinical accuracy, the differences between the clinical and the laboratory settings must be carefully examined.

#### Error sources

The accuracy associated with navigation based on preoperative MR/CT is independent of the accuracy associated with navigation based on intraoperative US. The main error sources associated with preoperative MR-/CT-based navigation are the image-to-patient registration process in a clinical setting, and the fact that the image maps are not updated to reflect the changing patient terrain as the procedure proceeds [24].

In contrast, intraoperative US volumes are acquired in the same coordinate system as navigation is performed. Image-to-patient registration is therefore not necessary, and a new US volume can be acquired to reflect the current patient anatomy whenever needed. However, navigation based on US is associated with its own error chain, in which the main error source is the US probe calibration process [25]. In addition, small variations in the speed of sound in different tissue may affect accuracy.

**Fig. 7:**
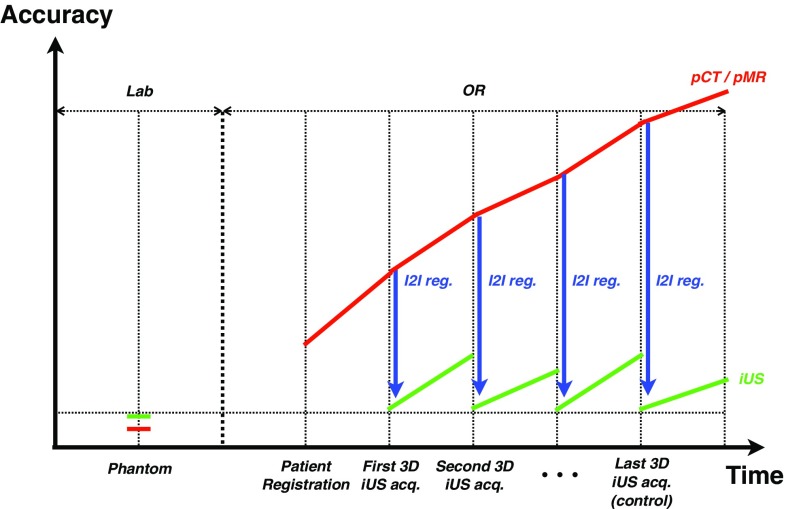
Navigation system accuracy (NSA) based on preoperative (p) MR (*red line*) and intraoperative (i) US (*green line*). iUS can be used to correct pMR using various image-to-image registration techniques (*blue line*)

A laboratory test of a system based on preoperative MR/CT using a rigid phantom will give the highest possible NSA (see Fig. 7, start of red line). A laboratory NSA above the accuracy required by the clinical application is a prerequisite as the NSA will be lowered when clinical factors are introduced.

**Fig. 8:**
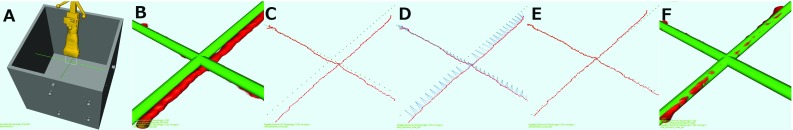
Automatic method for evaluating the accuracy in US-based navigation. **a** The phantom with a single wire cross in the middle of the water tank and a reference frame in the front, and a US probe imaging the wire. **b** 1-mm-diameter model of the nominal wire cross in green, and an US volume of the wire cross in *red*. A mismatch of about 1 mm can be seen **c** Centerlines of the nominal (*green*) and US (*red*) wire crosses. **d** ICP registration between the two centerlines, initial correspondence shown. **e** After convergence. US centerlines now match the nominal. **f** The displacement is equal to the NSA

The error due to probe calibration, the major source of error associated with US-based navigation, is included in the NSA results from accuracy evaluations using a rigid phantom in a laboratory setting. Furthermore, the surgeon judges the amount of tissue shift and deformation that is acceptable in a particular clinical case. A new US scan can be acquired whenever needed in order to have updated images for navigation (see Fig. 7, green line). As a consequence, the NSA found in a controlled laboratory setting will be valid in the clinical case given that navigation is based on a recently acquired US volume, either reconstructed or real-time 3D, and that the speed of sound used in the US scanner corresponds to the average speed of sound in the tissue. Preoperative MR/CT data can be corrected for anatomical shift and deformation using intraoperative US data and advanced image-to-image registration techniques [15, 17, 26, 27]. However, this is a challenging task introducing additional error sources. Therefore, the NSA associated with corrected preoperative MR/CT will not be as high as the NSA for US (see Fig. 7, blue lines). In addition, the independence between the two chains of error will be broken as NSA for MR/CT will be dependent on NSA for US.

The overall clinical accuracy of a navigation system is determined by the contribution from all the individual error sources involved [28]. The net effect will not be the sum of all the error sources, but rather a stochastic contribution from all the terms. Stochastically independent contributions are summed using the equation $E=\sqrt{\sum {(e_i)^2}}$.

#### NSA test method

As we have seen, the US-based NSA found in the laboratory using a phantom is valid in the OR as well as under normal conditions. An automatic, robust, flexible, and fast method for measuring the US-based NSA has been developed and integrated into CustusX. It can be used for substantially different US probes, and the phantom used is relatively easy to build and to characterize accurately. The technique, as shown in Fig. 8, consists of (I) a sweep with the US probe over a single wire cross in a water tank, (II) reconstruction of the US frames into a volume containing the cross, (III) segmentation of the wire cross from the US volume, (IV) extraction of the centerline of the segmented wire cross, and (V) registration of the resulting centerlines to a centerline representation of the accurately measured physical wire cross acting as a gold standard using the modified ICP algorithm [29].

This method was then used for assessing the NSA of a typical system setup with optical tracking (Polaris, NDI) and a US scanner (GE Vivid E9, 11L probe). The ultrasound probe was calibrated using a versatile calibration method (different probes, optic and magnetic tracking) presented in [30]. An evaluation of the calibration performance using varied metrics like point reconstruction accuracy (PRA) and calibration reproducibility (CR) can also be found in this paper. The overall clinical NSA was then estimated, based on a thorough understanding of the error sources that are present in a clinical setting.

#### Results

The laboratory NSA test results are presented in Table 1. The overall clinical NSA estimates are presented in Table 2.

**Table 1:** NSA measurements: mean (SD) in mm

Along wire cross, front to back (A-F2B), $n=3$:	1.12 (0.07)
Along wire cross, back to front (A-B2F), $n=3$:	1.15 (0.04)
Along wire cross (A), $n=6$:	1.13 (0.06)
Diagonal to wire cross, front to back (D-F2B), $n=3$:	0.75 (0.01)
Diagonal to wire cross, back to front (D-B2F), $n=3$:	0.94 (0.04)
Diagonal to wire cross (D), $n=6$:	0.84 (0.10)
All acquisitions, $n=12$:	0.99 (0.17)

**Table 2:** Overall clinical NSA estimates in mm

NSA using a phantom in the laboratory	$<$1.0
+ Calibration and position tracking of rigid surgical tool	$<$0.5
+ Interpolation 2D slice from 3D / tool cross indication	$<$0.1
= Overall NSA	$<$1.1
+ Sound speed uncertainty	0$-$2.0
+ Anatomical shift	0$-$10.0
= Overall clinical NSA	1.1$-$10.3

As can be seen from Table 2, it is possible to achieve an overall clinical NSA close to the laboratory NSA under favorable conditions, i.e., when the speed of sound used in the US scanner is close to the average speed of sound in the tissue imaged, and the US volumes are frequently updated. The need for updates can be determined by real-time 2D imaging. If these conditions are not met, the accuracy becomes poorer.

**Fig. 9:**
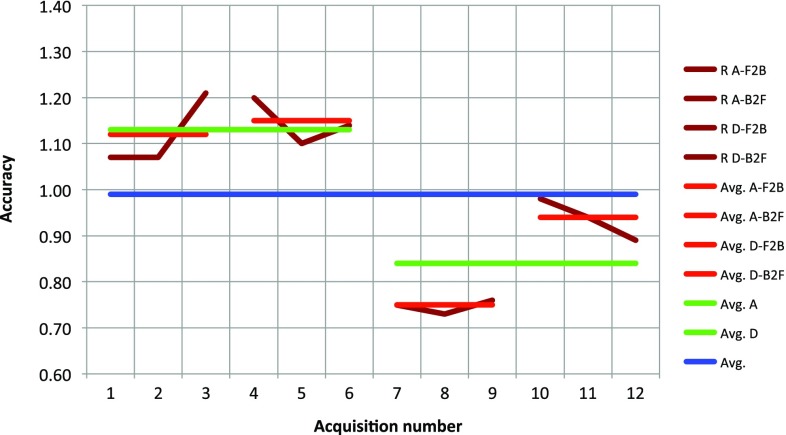
Accuracy as a function of acquisitions performed under various conditions; both individual measurements and various averages are shown. See Table 1 for an explanation of the different labels used, R = NSA

**Fig. 10:**
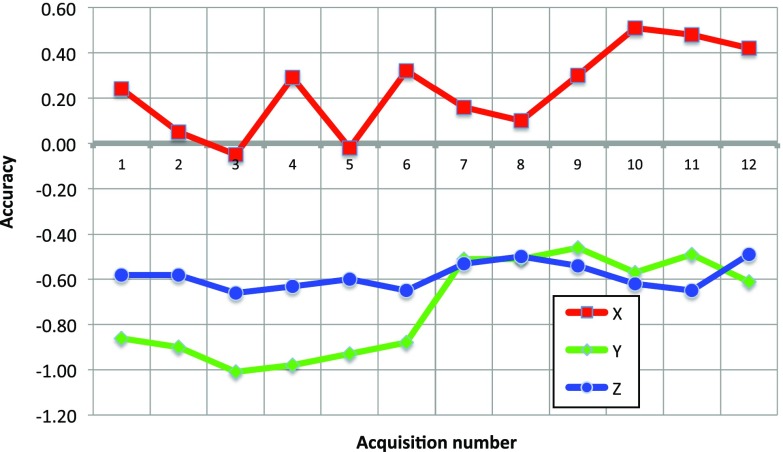
Accuracy components in the reference frame of the ultrasound scan plane as a function of acquisition number. The directions are elevation (*x*), azimuth (*y*), and radial (*z*)

The laboratory NSA data from Table 1 are displayed graphically in Fig. 9. The figure shows an average NSA below 1 mm, as well as high reproducibility (low deviations between acquisitions done the same way). However, the figure also shows variations between acquisitions performed differently, in terms of both probe movements toward versus away from the tracking camera (some variation) and probe movements along versus diagonal to the cross wire (some more variation). Furthermore, if we decompose the error vectors in the reference frame of the ultrasound sector/image, we see from the details disclosed in Fig. 10 that the errors in all three directions are relatively constant over all the acquisitions (possibly except for along vs. diagonal movement in the azimuth direction). This analysis is beyond the scope of this paper, but the measurements suggest that the accuracy varies in a systematic way, most probably due to suboptimal spatial probe calibration [18] and to a lesser degree temporal probe calibration errors. Nonetheless, if some of these errors can be corrected, submillimeter overall clinical navigation system inaccuracy should be possible under favorable conditions.

### System response

A good system response, i.e., high frame rate and low latency, is important for the operator. The frame rate is the rate of screen updates, while the latency is the total delay from an event occurs in the physical world to it is displayed on the screen.

#### System response test method

Frame rate is measured as the number of rendering operations per second. This is implemented as a feature and is displayed continuously on the screen with a 1-s averaging window.

Latency is measured by recording a scene containing a physical probe movement, the CustusX screen, and the US scanner screen using a 25-fps camera (Sony HDR-PJ740). The movie is then analyzed manually frame by frame in order to find the total latency.

Tests have been run on the system used regularly in the OR (Ubuntu 14.04, AMD FX-8350 $8\times 4$ GHz, GeForce GTX 6 GB, screen $2650\times 1440$), with a US scanner (Ultrasonix MDP) connected either via Ethernet/custom API (image size: $681\times 616$) or via a video grabber (Epiphan DVI2USB3.0)/OpenCV (image size: $1024\times 768$). Tracking (NDI Polaris) was enabled.

#### Results

Table 3 shows the frame rate measurements. The results indicate a base cost per rendering of 17, 33 ms per 3D view and $\ll$1 ms per 2D view and video view.

**Table 3:** Frame rate measurements, using combinations of 2D and 3D views covering the full screen

Layout	Frame rate (fps)	Render time (ms/f)
9 $\times$ 2D	60	17
1 $\times$ 3D+6 $\times$ 2D	20	50
2 $\times$ 3D+5 $\times$ 2D	12	83
Video + any	60	17

All views contained 2 volumes and a segmented model. The video view was used in various combinations with the same result. The 3D and video views use VTK rendering algorithms, while the 2D views use a custom renderer

Table 4 shows the latency measurements. No noticeable difference was found between the video grabber and the custom API; thus, the full average is shown here for US Video. As the tracker latency is about 70–100 ms [31] and the US scanner latency is measured to 171 ms, the additional latency introduced by CustusX is approximately 100 ms. Resolution is 40 ms due to the 25-fps camera.

**Table 4:** Latency measurements

Tool in 3D view	195 ms
US in video view	285 ms
US scanner	171 ms

The tool is displayed in the 3D view, the US video in a video view, while the US scanner is measured on its own screen for reference

## Clinical applications

CustusX has been used for research purposes in several different clinical procedures, of which some examples are presented in this section.

All studies were performed at St. Olavs University Hospital, Trondheim, Norway, and were approved by the Regional Ethics Committee Central. All study participants provided informed consent.

### Neurosurgery

**Fig. 11:**
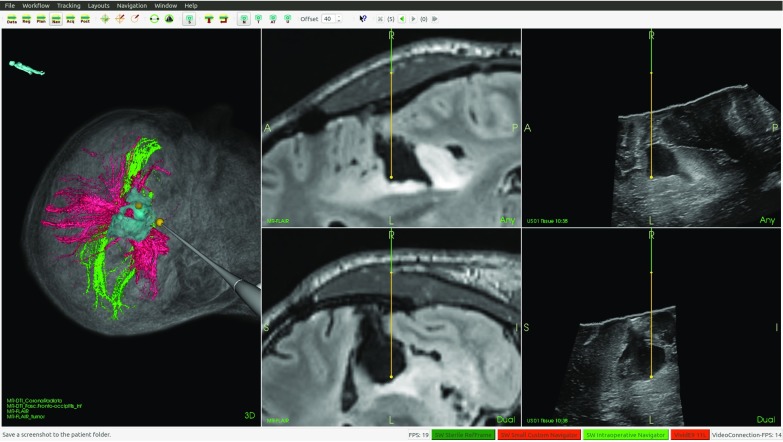
Intraoperative navigation using MR FLAIR and 3D US during resection of a low-grade glioma

**Fig. 12:**
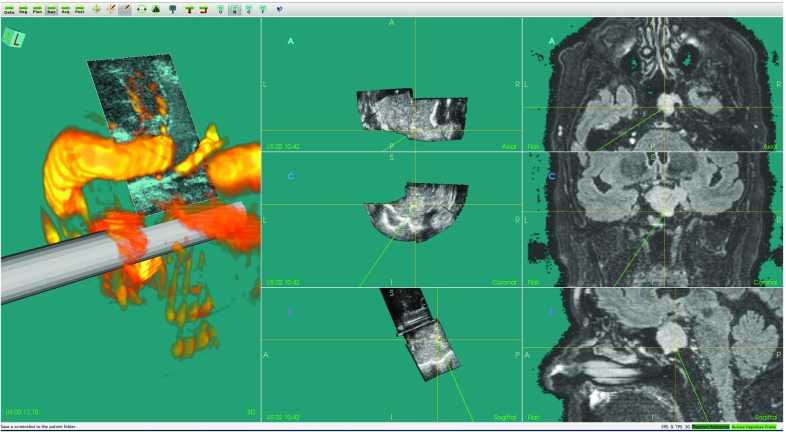
Navigation and intraoperative 3D US in surgery of a pituitary tumor. *Left column* model of the distal part of the prototype US probe with the real-time 2D US image plane shown at its correct location, and the volume rendering of a 3D US power Doppler volume. *Middle column* orthogonal image slices reformatted from a 3D US image volume. *Right column* corresponding orthogonal image slices reformatted from a preoperative MR volume

Accurate delineation of tumor borders throughout brain tumor resection (Fig. 11) and identification of feeding arteries in arteriovenous malformations (AVM) are examples of challenges where navigation technology and intraoperative ultrasound imaging can provide valuable information to the surgeon. In these and other similar procedures, we have used CustusX together with an optical tracking system and a US scanner to perform feasibility studies and evaluations of new technology. For most neurosurgical applications, we import preoperative MR images of the patient and perform an image-to-patient registration using fiducial markers or anatomical landmarks. During surgery, multiple freehand 3D US volumes are typically acquired for initial delineation of the target and to monitor the progress of surgery (Fig. 5). CustusX has been used to investigate the ability of 3D US to identify cerebral vasculature and vascular pathologies such as aneurysms and AVMs. Vascular structures seen in preoperative images, and intraoperative images can also be used to correct for brain-shift during the procedure [15]. For transsphenoidal surgery of pituitary tumors, the US imaging has been performed with prototype probes designed for the purpose of intrasellar imaging [32]. An example of 3D intrasellar ultrasound imaging is shown in Fig. 12. Other applications include data collection for more basic US research such as Doppler angle-independent 3D blood flow and velocity methods [33]. CustusX has proven to be a versatile platform for IGT research in neurosurgery, facilitating support for new ultrasound imaging techniques, prototype ultrasound probes, and development and evaluation of methods for brain-shift correction.

### Laparoscopic surgery

The laparoscopic resection of abdominal soft tissue tumors from, e.g., the liver or kidney are challenging surgical procedures. The aim of the procedures is to completely resect one or several lesions with a security margin and at the same time resect as little healthy parenchyma as possible. IGT and laparoscopic ultrasound can help localize the exact intraoperative location of the tumor, its relative position to important vessels and the boundaries of vascular regions making laparoscopic surgery more precise and safer [34, 35]. One of the challenges with navigation technology in laparoscopic surgery is the movements of soft tissue organs, which have to be accounted for in IGT [36].

We have investigated how CustusX can resolve these issues by providing navigation and US support. Preoperative images and extracted models of, e.g., the liver surface, tumors, and vessels have been imported. Navigated US has been made possible by mounting an electromagnetic position sensor close to the tip of a 4-way flexible laparoscopic US probe (Vermon, France). This has been used for both 2D real-time US and 3D US volumes reconstructed from these. Slicing in arbitrary directions and image fusion are used for visualization.

The system has been used with success in a number of laparoscopic procedures [37], where US gives exact localization information, while the preoperative data give a valuable overview with a lower accuracy. The main advantages of using a navigation system like CustusX compared to conventional laparoscopic US are the faster localization of the tumors, the better interpretation of the US images, and thus also in deciding the resection borders [34].

### Vascular surgery

During endovascular procedures, the vascular tree is accessed through percutaneous punction in the extremities or via small incisions in, e.g., the groin, and catheters and small flexible tools are maneuvered inside the blood vessels. For the interventional team to perform these procedures safely, accurate real-time imaging of the vascular anatomy is needed. Traditionally, this has been provided by X-ray fluoroscopy. This results in ionizing radiation to the patient and staff and also requires use of nephrotoxic contrast agents which can be a health hazard in patients with comorbidities such as diabetes mellitus and chronic renal disease [38]. 2D image modalities like X-ray fluoroscopy also make navigation in a 3D vascular structure a challenging task requiring highly competent operators.

**Fig. 13:**
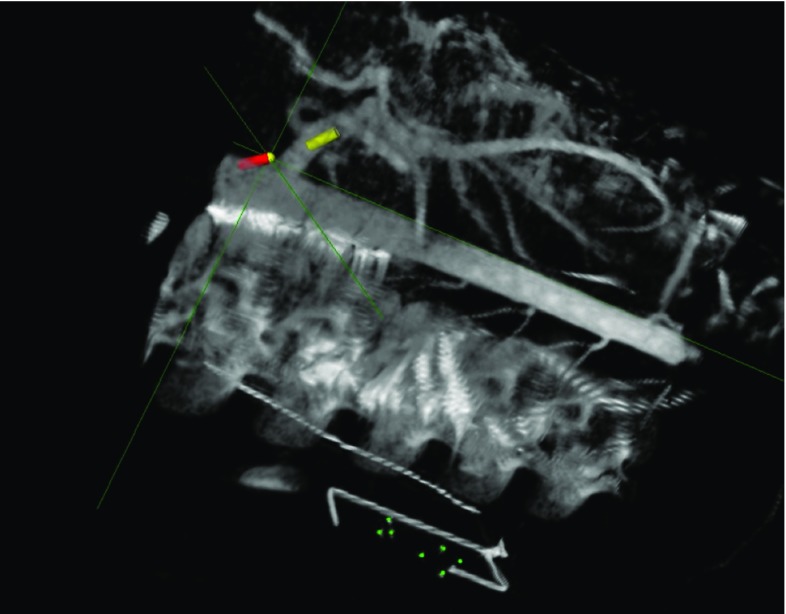
CustusX 3D view during endovascular navigation showing cone beam CT image volume and real-time position tracking of catheter (*red cylinder mark*) and guidewire (*yellow cylinder mark*)

We have investigated methods using CustusX as an image guidance platform during endovascular procedures. Preoperative CT and intraoperative cone beam CT were used as image input. Electromagnetic position sensors were integrated in catheter and guidewires and provided real-time position information for the tools. With this setup, we have been able to accurately and safely navigate the vascular anatomy of animal models [39] as shown in Fig. 13. Using a navigation platform like CustusX can potentially reduce the radiation dose and amount of contrast used during an endovascular procedure like endovascular aneurysm repair (EVAR) [40]. CustusX provides real-time navigation in an updated 3D anatomical model which also can facilitate complex endovascular procedures, like fenestrated or branched stent grafts for aortic aneurysms making them safer and easier to perform.

### Bronchoscopy

A challenge with bronchoscopes is the external diameter of 5–6 mm preventing advancement beyond the third-fourth division of the airways, in addition to the multiple bifurcations, making it difficult to localize the tumor. Even with fluoroscopy guidance, the biopsy diagnostic success rate for bronchoscopically non-visible tumors is as low as 15 % compared to 80 % for visible tumors [41]. Tracking of the position of the bronchoscope and associated tools increases the success rate up to 80 % for peripheral targets [41]. In addition to an increase in the efficiency in lung cancer staging, tracking of tools also provides new possibilities for procedure documentation, e.g., of the biopsy positions.

CustusX has been used as a guiding system during guided bronchoscopic examinations. The airways and their corresponding centerlines are automatically segmented from the CT images using a method integrated in the navigation platform [13]. Tumors and lymph nodes, which are the targets for the staging procedures, are visualized in the navigation scene.

**Fig. 14:**
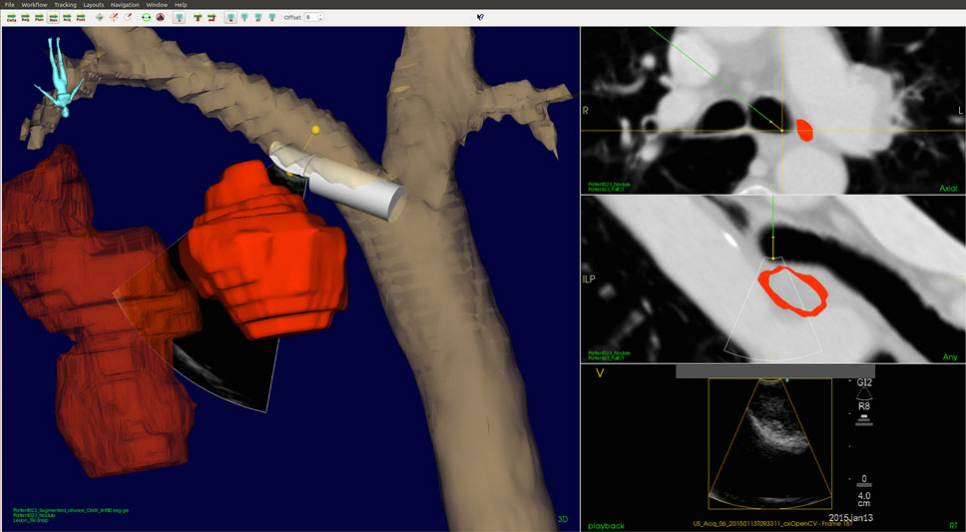
A navigation scene during EBUS-TBNA. *Left* 3D representation of the segmented airways, EBUS scope, lymph node, and tumor. *Right* Axial view of CT (*top*), view of CT corresponding to the US sector (*middle*), and US (*bottom*)

In endobronchial US transbronchial needle aspiration (EBUS-TBNA), a linear US probe is positioned on the tip of the bronchoscope, allowing visualization of anatomical structures outside of the tracheobronchial wall. Using EBUS-TBNA in combination with CustusX allows fusion of US and CT images, which is likely to improve the efficiency and accuracy in localizing the designated target for needle aspiration. Figure 14 shows the CustusX navigation scene during EBUS-TBNA.

In lung navigation with CustusX, an automatic image-to-patient registration is used, which matches the trajectory of the bronchoscope tip with the centerline of the airways extracted from the CT [42]. The algorithm, a modified version of an ICP algorithm, utilizes in addition to the positions the orientation of the bronchoscope and the running direction of the CT centerline in the alignment process. Positions of the bronchoscope tip for the registration procedure can, for instance, be acquired during the typical anesthetic procedure at the initial part of the examination, thus not adding any extra time or work load compared to a regular bronchoscopy.

## Discussion

We have presented the CustusX navigation platform for IGT and described the system architecture, typical workflow, and quality process. Validation of critical system parameters such as navigation accuracy, latency, and frame rate has been presented. We have described the use of CustusX in several clinical areas and showed the versatility and the potential of the platform.

### NSA and system response

Several factors are important in order to use a research system in the OR, where accuracy is one of them. Either the operator must have an accuracy below what is needed in the procedure at hand, or the data must clearly be marked as inaccurate. We have shown that the overall NSA is $<$1.1 mm for ultrasound-based navigation, which is acceptable for the procedures we have studied.

Furthermore, the system must be responsive, i.e., frame rates about $>$20 fps and latency about $<$200 ms for navigation and video. While the typical frame rate for CustusX of 20 fps is acceptable, the video latency of up to 285 ms will be noticeable for fast movements. However, this has proved to be sufficient during normal movement.

### Software: quality and architecture

Software stability and quality are also important issues in the OR. Systems that seem to work well in controlled conditions of the laboratory are not necessarily stable enough to be used in the OR, where rapidly changing requirements due to the procedural flow can cause software to fail in unexpected ways. A good quality process is therefore crucial. Over the last years, we have spent a considerable amount of time and effort on the quality system with the result that CustusX can now be used for full day procedures without problems. It must be remembered, however, that there will always be a fundamental difference between research and commercial systems: A research system should be in front of the development and bring new features into the clinic faster than commercial systems which concentrate on stability and ease of use.

The architecture of CustusX has been changed several times over the years. The latest refactoring into an OSGi-based plugin framework was an important and successful milestone in the CustusX development. This refactoring was a major task, but has greatly simplified the architecture and module dependencies. Startup and shutdown of the system are now trivial because of the temporal nature of the services. Extensions of the system through plugins have also become simple because of the standardized framework and the similarity to other systems that also use CTK [1, 3] . As much of the new code are written in plugins, the overall complexity of the system increases more slowly than before and it is easier to isolate code of varying quality or with a large degree of specialization. The architecture is also suited for toolkit-like usage. Several customized applications are currently being developed using CustusX as a basis.

### Interaction and change in practices

The CustusX development process revolves around the needs of the clinic and clinical studies, and we try to avoid the drive to implement new technology per se. This process is enforced by maintaining a tight cooperation between software developers, technological and clinical researchers through informal discussions, weekly meetings, frequent attendance of technological researchers in the OR, and joint attendance at national and international conferences.

The integration of IGT with the use of intraoperative US requires the introduction of high-tech equipment in the OR, changes and adaption of clinical practices, and a good understanding of US image interpretation [35]. We therefore organize clinical courses to spread technology and clinical methods.

### Other platforms: comparison and cooperation

There are several other platforms that operate in the same field as CustusX. The two major ones are 3D Slicer and MITK. 3D Slicer is application centered, but extensible via plugins, while MITK can be extended by plugins and embedded in new applications. Both are generic platforms for medical image analysis research.

For comparison with CustusX, 3D Slicer can be combined with the PLUS framework to create an IGT platform. MITK-US gives MITK many of the same features that are found in CustusX and is an example of how a generic system can be converted to IGT. The advantages of both of these systems are that they draw upon the resources of platforms with large feature sets and a well-defined quality process. CustusX is dedicated to IGT, which means that it is ready for use in the OR, the user interface and features are tuned toward intraoperative use, and it has a complete feature set for intraoperative US. Each of these systems have their strengths and some of the functionality is overlapping. The implementation is also shared to a large extent because they are all built upon the same open-source libraries. As part of the CustusX development, we have started to integrate with the PLUS framework through OpenIGTLink as an optional tracking implementation, and we cooperate with both through the CTK library.

We believe that the best way to cooperate is through common libraries and interfaces. This way every platform can develop freely while sharing the majority of the code. A multitude of applications and platforms also strengthen the common libraries they are built upon through different usage patterns.

### Open source

CustusX has been developed and used for more than 15 years, but only recently published as an open source. The publication of the source code comes as a result of a shift in the software vision in our institution. Earlier, code was seen as property and a tool used to produce knowledge. Now, source code is seen as part of the knowledge.

There are several important points supporting open-source software: Code is an integral part of the produced knowledge; thus, the credibility of scientific results and published papers is strengthened. It also contributes to reproducible research results as others can perform experiments using the same tools. Better access to necessary tools can also trigger more research in the field. Public code will be viewed by more people, displaying strengths and weaknesses. Ultimately, this will lead to better code. Most of our research projects are funded by public sources; thus, the results should be available to the public. Lastly, the ultimate goal of doing research is to contribute to the global body of knowledge.

By publishing our code, we declare ourselves as part of the open-source community: All knowledge should be open, including papers, software, and data. Our intent is to continue this path by also publishing parts of our extensive databases of clinical image data accumulated over the years. While the open-source strategy applies to our own work, software partially owned by other companies may still be kept proprietary.

### Future directions

CustusX will be continuously developed and improved in the years to come to meet new research requirements and adapt to new technologies such as new tracking devices, new ultrasound scanners, 3D and wireless US probes, and flexible tools. With the current platform in place, CustusX will be used for different purposes including development of new applications customized to specific clinical procedures, prototyping of new modules for validation in the OR then possibly being integrated into commercial systems, and in basic research where new ideas need an implementation in order to be evaluated.

## Conclusion

CustusX has matured into a useful tool for research in intraoperative navigation and ultrasound, with a stable code base and software quality process. The developers will continue to contribute to the medical open-source community through both CustusX and its dependent libraries. We hope and believe that CustusX will be taken into use directly and as a platform in the years to come.

## Footnotes

1 http://www.usigt.org.

2 http://www.custusx.org.

3 http://www.normit.no.

4 http://www.qt.io.

5 http://vtk.org.

6 http://itk.org.

7 http://eigen.tuxfamily.org.

8 http://dcmtk.org.

9 http://www.itk.org/Wiki/MetaIO/Documentation.

10 http://www.videolan.org/vlc.

11 https://github.com.

12 https://github.com/philsquared/Catch.
